# Observations on the Use and Abuse of Emetics

**Published:** 1832

**Authors:** Daniel Drake

**Affiliations:** Cincinnati


ART. III.—*Observations on the Use and abuse of Emetics.* By DANIEL DRAKE, M. D.

For some time past, emetics have been gradually falling out of use in the United States, and I believe likewise in Europe. As they are *unpleasant* medicines, it would be desirable to dispense with them altogether; but this, I apprehend can not be done without injury to the resources of the profession. I am, even, inclined to believe, that many physicians have already gone too far in their neglect of these ancient and powerful remedies; and I propose to recall the attention of the readers of this Journal to their value.

### I. OF THE CAUSES OF THEIR DISUSE.

These seem to be chiefly the following:

1st. The epidemic yellow fever of 1793, in which they were not found beneficial, and were generally replaced by cathartics, particularly the well known composition of calomel and jalap so strongly recommended by Dr. Rush; at that time, and for many years afterwards the most authoritative medical man in America.

2. The popular writings of Dr. Hamilton, in favor of cathartic medicines.

3. The recommendation by Mr. Abernethy, of the blue pill and rhubarb as means of correcting a morbid state of the digestive functions.

4. The gastro-enteritic doctrines of Broussais, with their prohibition of almost every remedial means, except abstinence, the lancet, and leeches.

5. The increasing tendency of the profession, to view the morbid secretions present in the alimentary canal, as the mere signs and effects of irritation in the solids, and of themselves quite harmless.

Now it is quite possible, that these and other causes may have brought emetics into *unmerited* disrepute; for, in the profession, as in social life, the multitude are prone to join in a clamor, however unfounded; and the majority of its members find it much easier to ascertain what is fashionable than what is just and proper. That they have thus combined in reference to the use of emetics, may, I think, be safely afflrmed. As far as they have reasoned at all, it has been from the abuse of this class of remedies, or from theories that have imposed an *a priori* prohibition. But practical medicine is, essentially, an experimental science, and should place its chief reliance on trial and observation.

### II. Of the Modus Operandi of Emetics.

Vomiting is a natural remedy, and presents us with one of the best marked examples of a *vis conservatrix et medicatrix*, to be found in the whole circle of sanito-morbid actions. The cases indeed are not a few, in which its occurrence is followed by a healthier state of the system, although not produced *secundum artem*. When thus excited, its effects are by no means limited to simple evacuation of the stomach; but extend to almost every organ and tissue of the body.

The operation comprises three successive stages, in each of which the vital properties and functions are differently and powerfully effected.

1. *The Stage of Nausea.* There can be no mistake in the observation, that the first effects of an emetic medicine are debilitating. The pulse becomes feeble, empty and frequent; the skin pale and cool; the muscles of animal life languid, and the ligaments relaxed, so that stiff and diseased joints may be moved to a greater extent, and with less pain than before; the organs of sense are blunted in their susceptibility; the faculty of observation impaired, and the intellectual functions crippled.

Thus it is clear, that the brain and heart, with the irattached systems of nerves, muscles, and blood vessels, are in a state of reduced excitement. It is held by some, however, that this condition is preceded and accompanied by inflammatory irritation of the mucous membrane of the stomach. But this is, certainly, a gratuitous opinion. That such a state of that membrane

*may* consist with this pathological condition of the organs, cannot be denied; but that inflammatory irritation of the stomach, is not necessary to the production of nausea, and does not in general occur, may be inferred from various facts and considerations.

1. Nausea may be produced by several causes which cannot be suspected of exciting nervo-vascular irritation; as for example, the copious use of tepid water, titillation of the fauces; offensive odours; swinging, whirling, carriage-riding, sailing, and other gyratory, or undulatory exercises; blood-letting, and concussion of the brain; to say nothing of weak infusion of ipecacuanha and other mild vegetable emetics which seem totally destitute of the properties necessary to the direct production of inflammatory action.

2. The stomach may be severely irritated and inflamed by various stimulants, such as cloves, cinnamon, or cayenne pepper, without exciting nausea.

3. Many cases of chronic gastritis, known under the name of dyspepsia, are unattended with nausea.

4. The time which follows the administration of an emetic medicine, before nausea and vomiting are induced, is, in general, too short to admit of the formation of an inflammatory irritation.

5. The symptoms attendant on this state, are not those of the nausea connected with marked gastritis.

6. The state of the stomach immediately on the subsidence of the nausea, is so sound and comfortable, as to discountenance the idea of a previous inflammatory orgasm.

Thus I am led to conclude, that nausea is a *peculiar* condition of the stomach, necessary to the natural function of vomiting; which may, or may not be associated with active irritation, according to the nature of the agent that produces it; and which, when occasioned by the ordinary therapeutic means, is rather asthenic than sthenic. In short, that the stomach is in harmony with the other organs of the body.

It may be said, however, that during nausea, there is a great secretion of mucus and saliva; but such increase, by no means argues inflammation—perhaps the reverse—for there is increased perspiration, while no appearance indicates inflammatory congestion of the skin. In a fit of terror, moreover, there is augmented intestinal secretion; and in hysteria, from the same cause, the kidneys pour fourth copious streams of urine; but neither the bowels nor kidneys manifest signs of inflammation. In both cases, the increased secretion results from an affection of the nervous system, and the increased mucous, salivary, bilious and perspiratory secretion, from nausea, is manifestly of the same kind.

2. *Of the Vomiting.* The condition just described of the gastro-pharyngeal membrane, precedes and excites the convulsive movements of vomiting. Whenever it takes place, the brain is necessarily involved, as no other organ could combine the muscular apparatus of respiration, and the muscular tunic of the stomach, œsophagus and pharynx, in a simultaneous and co-operative movement. The effects of this extensive and violent muscular contraction are decisive. The abdominal and pelvic viscera, are strongly compressed and agitated; and the contents of the stomach, duodenum, gall-bladder and hepatic ducts are brought up. The lungs are alternately compressed and dilated to an unusual degree; the descending aorta is mechanically obstructed by the viscera, and a greater quantity of blood is determined into the arteries of the brain, which is thereby disturbed in its functions; the blood of the portal viscera and the convulsed muscles, is returned more rapidly upon the heart, whose movement becomes hurried and irregular; lastly, the spasms of the muscles of the tongue and throat agitate the larynx and glottis. Inverted muscular contraction occasionally takes place, quite down to the ileo-cæcal valve, but more commonly the increased action is progressive, and leads to purging. In the system of animal life, the contraction sometimes extends to the muscles of the extremities, especially the lower, in the form of cramps and spasms, and, in a few cases of hyper-emesis to the whole body, in the form of dangerous convulsions.

Such are the prominent circumstances of the second stage, which is, emphatically, a state of muscular excitement; to the production of which, however, an active irritation of the stomach is not necessary, as we see the same kind of convulsions, before death, from the loss of blood.

III. *Of the Sweating Stage.* Sooner or later after the commotions of the second stage subside, a state arises, which is characterized by sleep, a full, soft and slow pulse, general perspiration, and acuter and healthier sensibilities both of mind and body.

These three stages may always be recognized, when an active emetic is administered to a person in health or but little indisposed. It is easy to perceive in them many analogies with the three stages of a paroxysm of intermittent fever—that of depression or chill, of febrile excitement, and of secretion.

Vomiting, from a therapeutic agent, may in reality be made a disease, as pervading and extensive as intermitting fever; but the second stage affects the muscles, chiefly, instead of the blood vessels, and the fit is not repeated unless the remote cause be reapplied. The first effect of this remote cause is the production in the gastric mucous membrane, of an action, *sui generis*, not necessarily accompanied with inflammatory orgasm, which springs the muscular tunic of the organ and the muscles of respiration, into strong contraction, and by dependence of function or sympathy, that is, anatomically or physiologically, alters the vital properties and functions of the whole body, in many cases obviously improving the condition of both.

If the medicine be administered in such doses as to produce nausea, only the powers of the heart and brain are more deeply depressed than if vomiting be excited, and the subsequent state of the vital properties is much less active. Nausea is, indeed, a sedative, but vomiting has many claims to be regarded as a stimulant, and, therefore, an active and speedy emetic debilitates less than protracted nausea, purging or venesection.

To the absorbent system, both nausea and vomiting are stimulants, for collections of pus and serum begin to be absorbed during the former, and often rapidly disappear under and after the cessation of the latter.

Thus an emetic, beyond almost every other therapeutic agent, affects the whole system; acting as a sedative, a stimulus, and an evacuant—renovating the vital properties—disturbing, but ultimately equalizing, the currents of the circulation—promoting absorption, and restoring the suspended secretions.

### III. Of the application of Emetics to the cure of Diseases.

It would be a great mistake to suppose, that the good effects of emetics are limited to original diseases of the stomach, or depend entirely on the gastric evacuation which they produce; and equally erroneous to infer, that every malady in which they do good, is seated in that organ. Every primary gastric disease, does not call for vomiting, and many disorders which do not affect the stomach, are mitigated or removed by that operation. A disease is cured, when the morbid function in which it consists is made to cease, either by reducing the powers of the organ, by introducing a new action, or by transferring the scene of operations to another part. Whatever can accomplish either of these, is remedial. Emetics to some extent, are capable of effecting the whole. In what cases they will sncceed or fail, can only be determined by experiment. I shall proceed to enumerate the principal maladies, for which I have found them beneficial.

In the Anorexia with furred tongue and bitter taste, head-ache, back ache, and muscular debility, so often present in spring and summer, and known among the people under the name of "bilious habit," "a bilious attack," and "foul stomach" emetics generally give immediate relief.

When Cholera is impending, an emetico-cathartic, is frequently attended with the happiest effects. The artificial disease is made, as it were, to take the place of the other, and when it subsides, the patient is in general, speedily restored, especially if the operation be succeeded by an opiate and sudorific.

In Diarrhœa, emetics of ipecacuanha are often followed by signal mitigation of the increased peristaltic action; by determination to the cutaneous surface, and by consequent diminution of mucous secretion. They frequently restore suspended hepatic secretion, a state which produces diarrhœa, and thus effect a cure of the latter.

In Dysentery. Emetics and Emetico-Cathartics, are among the most efficient remedies in this malady; though its modern name —*colitis*—may be sufficient, with a part of the profession, to lead to their prohibition. When the accompanying fever is acute, venesection should be premised; and when the tormina are excruciating, some preparation of opium, should precede or accompany their exhibition.

In chronic Hepatitis, emetics are often followed by the happiest effects. The liver is emulged, both its secretory and transmitting functions improved, the sympathetic affections of the stomach subdued, and the deep and distressing nervous irritation, attendant on the visceral derangements, more signally allayed, than by any other means that I have ever employed.

Jaundice and Gall Stones. When Jaundice is attended with hepatic inflammation, or passing gall stones have excited that disease in the duct, blood letting is requisite; but this step being taken, or judged unnecessary, emetics are of great benefit; being frequently followed by a free and regular discharge of the contents of the gland and cyst, and an immediate abatement of all the symptoms. To be effective, they should be of the more energetic kind, and the vomiting should be violent and protracted.

Among the varieties of Colic, cases are often met with, to which they are well adapted; as, for example, such as are excited by indigestible substances.

Professor Hosack has strongly recommended them in Constipation. When that symptom is not attended with developed inflammation, their union with cathartics renders the latter far more effective.

Enlargements of the Spleen, from intermitting fever, require an occasional emetic.

Dyspepsia. The practice of administering emetics in this disease, has the sanction of an ancient and varied experience. At the present time, fashion regards it as simple chronic gastritis. I am convinced, however, that the majority of cases are either unattended with inflammation; or, that a slight degree of gastritis does not contra-indicate vomiting—probably both. Dyspeptics of a sluggish and phlegmatic habit, are most benefited by emetics, and, among this class, those who lead sedentary lives, will derive the greatest advantage. The sensibilities and digestive powers of the stomach, the functions of the skin, and the operations of the nervous system of animal life, are signally improved by an active emetic. In many cases, however, it is necessary to premise venesection or epigastric cupping.

Inflammations, from cold of the Pharynx and Tonsils, (*Cynanche pharyngea et tonsillaris*) are treated with great success by emetics. A single blood letting, followed by active vomiting, will, in most cases, discuss the inflammation, and save the patient from a tedious suppuration—a result, that might, in fact, be anticipated, from the intimate sympathy, so observable between the pharynx and stomach, and the striking effects which emetics excite in the former.

In diseases of the Larynx, Trachea, and Lungs. The beneficial effects of emetic medicines in most of these maladies, are generally acknowledged by the profession.

In Pertussis they are known to be among the most efficacious palliatives.

In Croup (acute Laryngitis of children) their reputation is established. Administered in the forming stage, they frequently arrest the disease. When it is fully established and violent, they require blood letting and the tepid bath to secure a free operation.

In Catarrh, or irritation of the entire naro-pulmonary mucous membrane, whether epidemic or sporadic, their effects are eminently serviceable; especially if preceded, in violent cases, by blood letting, and followed by diaphoretics, and copious dilution.

In chronic Laryngitis of adults, they are useful palliatives; in the acute, they are curative.

In Bronchitis, both acute and chronic, they produce excellent effects; promoting copious secretion and contributing to a free expectoration of mucus.

In Pneumonitis and Pleuritis, they are valuable, the lancet of course being first adequately employed.

In the whole of these affections, tartarized antimony, in the form of pills, in large doses, with or without opium, produces effects that go far to justify the exalted eulogies of Thommasini and other Italian physicians.

In Tubercular Phthisis an occasional emetic is productive of mitigation; of course nothing more should be expected.

In Hæmoptysis I have excited nausea with much advantage; and there are respectable authorities for vomiting, to which, however, I have not been accustomed to resort.

In Asthma, not arising from organic læsions, they often prove beneficial in an eminent degree, especially when the dyspnœa is occasioned by a bilious or acid state of the stomach.

Various affections of the face and head require the use of emetics.

Ophthalmias, the offspring of malaria, or of dyspepsia, are frequently mitigated by them.

In Amaurosis they are useful, provided the brain be not engorged.

Ordinary sick head-aches are greatly relieved by them, if taken at the commencement of the fit, and the feet be bathed in warm water.

In some varieties of Neuralgia, they have been found beneficial; and often give immediate relief in that pseudo-neuralgic affection, the "jaw-ache," from decayed teeth.

In cases of Arachnitis (*Hydro-cephalus Infantum*) attended with but little fever, and arising from gastro-intestinal irritation, they are valuable remedies.

In Apoplexy, connected with gastric repletion, they may follow blood-letting with great advantage. When the patient is sanguine and plethoric, and the original irritation is in the brain, they are contra-indicated.

They are well adapted to many cases of Chorea, a disease in which the appetite is often both excessive and perverted.

In at least two varieties of Epilepsy, they are valuable.—1st. That from indigestion, occurring within the true dyspeptic period, that is between the 18th and 36th year; and, secondly, that which arises from intemperance in its earlier stages, and immediately follows a debauch.

When Hysteria, as often happens, is occasioned by gastro-intestinal irritation, they frequently giveim mediate relief, after the usual anti-spasmodic medicines have been administered in large doses without effect.

In Mania and Monomania, they are often beneficial; but the selection of cases for their employment requires the exercise of a discriminating judgment.

They are proper in a greater number and variety of cases of Hypochondriasm, effecting the expulsion of large quantities of bile or acid, and dissipating in a single hour, the deepest intellectual gloom.

In Mania a Potu, they are of unquestionable utility, but if I mistake not, the profession, at large, have not realized all that has been recorded in their favor by Dr. Klapp. I certainly have not myself, and when the constitution is shattered, they may do harm.

In many cases of Rheumatism, their efficacy is very great. I have repeatedly seen a powerful vomit with or without previous blood-letting, put a stop to a rheumatic attack, which, to all appearance, would otherwise have been protracted and severe. Many persons are subject to what they call pain in the bones—an affection of the periosteum. I have seen it very generally removed by an emetic.

Of their value in Dropsies, no one who gives them a full trial will entertain a doubt. Indeed, no other therapeutic agent more powerfully excites the absorbent system. When a decided phlogistic diathesis exists, they should be preceded by blood-letting. When the effusion is the consequence of organic disease, obstructing and deranging the venous circulation, their good effects are of course more limited. It is to persons of the lymphatic temperament that they are most beneficial.

In Chlorosis they are admirable remedies, preparing the system of the patient for the Bark and Chalybeates, and enabling those tonics to exert a power which they could not otherwise manifest. To the cure of Leucorrhœa they are nearly as applicable; except when vomiting is contraindicated by prolapsus uteri.

In Puerperal fever, I have repeatedly witnessed their salutary effects. In this, as in many other maladies, they are more beneficial when united with cathartics, than when given alone.

Most of the Eruptive fevers are moderated by emetics. Erysipelas is sometimes cut short by their active operation.

Small Pox and and Varioloid affections are mitigated by them, if administered during the eruptive fever.

In Measles they are important remedies. When the lungs are engorged, and the cutaneous efflorescence is tardy in appearing, an emetic and the tepid bath, with or without blood-letting, according to the degree of arterial excitement, will seldom fail to establish it completely; while to meet the sinister symptoms, which sometimes follow the recess of the eruption, they are of equal value.

They are perhaps more efficacious still in the different varieties of Scarlatina, particularly when attended with dangerous ulceration of the throat.

In the treatment of Intermittent fever, they possess great efficacy. Given before the chill, they not unfrequently ward it off, and shorten the paroxysm. Administered in the hot stage, in connexion with refrigerants and cathartics, they moderate its violence, and prepare the system for an early and salutary resort to the Bark or its preparations. In protracted cases, an emetic, in connexion with an opiate, at the access of the paroxysm, will sometimes cure the disease. In congestive intermittents, they are useful; but should be administered in connection with opium sinapisms, the warm salt bath, and other cutaneous stimulants.

In the higher grades of Remittent autumnal fever, Emetics are contraindicated, at least until copious blood-letting has been premised. When bile was absurdly regarded as the cause of this fever, Emetics were held to be essential to its cure: Now that it has become fashionable to consider it as a gastro-enteritis, and the bile as a *harmless* product of duodeno-hepatic irritation in the solids, Emetics are on theory pronounced to be pernicious. Were I to rely on the results of my own observation, I should say, that they are sometimes pernicious, and sometimes unnecessary, but often beneficial The best, I think, is tartarized antimony, which should be given, so as to excite protracted nausea, 2; in connexion with refrigerants and cathartics, as the nitr. pot. sulph. magnes. and Supertart. pot. 3 in the exacerbation.

In Typhus and typhoid fevers, I have repeatedly seen Emetics do much good—diminishing the acrid heat and parched dryness of the skin, increasing the secretions of the mucous membranes, lessening the frequency of the pulse and rousing the patient from coma.

In the Epidemic denominated Pneumonia Typhodes—a pulmonary inflammation associated with a typhus diathesis of the general system—emetic medicines were, on the whole, the most efficacious that I saw employed. In large doses, so administered as not to operate, they did much good, while full vomiting seldom failed to produce a decided mitigation of the cough and dyspnœa, and to improve the condition of the pulse. When the lancet was of doubtful propriety, emetics generally answered every reasonable expectation.

These are the principal diseases in which I have employed Emetics with advantage; but there are many others in which a preliminary, or an occasional vomit, is of decided benefit. It is not difficult to comprehend how this happens.

When the stomach is oppressed by morbid secretions, either mucous, acid or bilious; or, its vital properties are obtunded or perverted, no medicine will produce its characteristic effects.—In such a condition of the organ, sudorifics, narcotics, tonics, expectorants, diuretics, emmenagogues, and even cathartics, are seldom followed by decided advantage; but, preceded by a vomit, which completely empties the stomach and augments its excitability, their action is energetic. It is like deterging and inviting blood into the skin, before the application of a blister. Now every physician is aware, that in many diseases, the stomach, which is to receive most of our medicines and transmit their impressions to distant organs, is exceedingly apt to become the seat of morbid accumulations. These matters, it is held by many practitioners, may be effectually removed by cathartics, to which, on several accounts, they give the preference. I have satisfied myself, however, that a cathartic may operate copiously, and yet leave the stomach clogged with recremental matters or morbid secretions; and, I believe, moreover, that vomits augment the sensibility of the organ, more than purgatives, and, therefore, on both accounts, are in numerous cases a better preparatory means.

The shock which Emetics impart to the whole system, is another reason why, as an occasional remedy, they prove beneficial in various diseases, (to the perfect cure of which they are inadequate,) although such complaints may not present any symptom of gastric derangement. I am aware, that this shock is regarded by certain physicians with dismay; but their apprehensions are the offspring of theory rather than practice. It remains to be shown, that diseases in general, consist in inflammatory irritation, especially seated in the gastro-intestinal mucous membrane; or that, if possessing this character and locality, they can only be cured by abstinence and sanguineous abstractions. Of the value of these therapeutic means, I entertain an exalted estimate; but cannot concede to them the right to exclude other remedial measures. If inflammatory irritations may arise, when the sum total of the vital powers, is below the standard of health, it follows, that they may continue under a simply reducing method of treatment, however far it may be carried. They who rely on mere reduction, will have many tedious and fatal cases, which might have been averted or cured, by a well timed resort to such things as agitate the system, modify its vital properties, and introduce new actions. As a general proposition, when morbid actions have been reduced to a certain degree, they may be superseded by something which makes a strong impression. Such an impression is not of necessity, incompatible with the well being of the constitution. In fact, our organs, like plants and animals, flourish under agitation, and become healthier and firmer in proportion as they are, within certain limits, subjected to hard treatment. I have seen nothing to convince me, that energetic and agitating methods of treatment, impair the constitution; on the contrary, I believe that bloodletting, emetics, cathartics, narcotics, the cold effusion, and tonics, employed in such manner as to carry rapid and decisive changes into the various organs, frequently preserve them from the wasting influence of chronic maladies; and by renovating the vital properties, give renewed and healthy activity to the different functions. Thus it is, I think, that we may comprehend, how emetics are safe and salutary in a large number of diseases, their application to which may in reality be regarded as empyrical.

### IV. Of the Precautions necessary in the Use of Emetics.

Although vomiting is a natural function, it cannot be excited under all circumstances, to any extent, or by every kind of nauseant, with impunity. Hence emetics are perpetually misused; and often do harm instead of good, to the great injury of their character.

A plethoric state of the blood vessels, with powerful action of the heart, contraindicates their use; and when this condition of the general system, is connected with inflammation of any of the important organs or tissues, they may be extremely injurious. Thus in high grades of autumnal fever, and in acute gastritis and arachnitis, they are decidedly improper. The predisposing remedy, in these and other specimens of intense phlogistic action, is copious bloodletting; immediately after which, if vomiting should be indicated, it may be brought on with facility, safety, and good effect—especially if tartarized antimony be selected and so administered as to excite protracted nausea. In most of these cases, it is advisable to combine the medicine, with a cathartic, such as calomel and jalap, or the sulphate of soda or magnesia; when the effect is invariably more salutary. Indeed, a saline emetico-cathartic, after a full bleeding, is one of the most powerful means, which the profession can wield against an active inflammatory diathesis; and often accomplishes that, which mere bloodletting could not effect, on account of the tendency of that remedy, when often and quickly repeated, to generate a state of dangerous nervous irritation.

The union of cathartic with emetic medicines, has another advantage. It tends to prevent cramp of the stomach. Hence, when that morbid state arises without purging, a cathartic injection is one of the most prompt and successful remedies in our power. But more of this presently.

Another auxiliary and corrective measure, in reference to Emetics, is the administration, after the vomiting has ceased or in its latter stages, of a dose of opium or one of its preparations, combined with a diaphoretic. By this practice the irritability of the system, accumulated and rendered morbid by the emetic, is subdued; the functions are tranquillized; the pulse rendered full and soft, and perspiration promoted. The patient enjoys refreshing sleep, and, generally, awakes with improved feelings and better health.

It is an abuse of Emetics to administer them very frequently. They first render the stomach irritable, and then inflame it; especially if venesection be not premised, and cathartics and narcotics interposed.

Certain incurable organic maladies of the viscera, contraindicate the use of Emetics. When the lungs are cavernous, and considerable branches of the pulmonary artery are liable to erosion or ulceration, they may bring on hæmoptysis. When the ventricles of the heart are affected with passive dilatation, or the great arteries of the chest are aneurismal, they are dangerous. When the stomach is the seat of ulceration, or the pylorus is scirrhus, they should be withheld.

Persons of a phlegmatic temperament, are sometimes injured by Emetics, particularly the antimonial. They prostrate the muscular power, both in the stomach and the apparatus of respiration, and excite deep and distressing nausea, with paralysis, rather than convulsion, in the muscles concerned in vomiting. When persons of this temperament take Emetics, they frequently require stimulants, such as a strong bitter infusion, weak spirit and water, powdered mustard thrown into hot water, or a moderate draft of paregoric, in the same vehicle.

A nervous temperament, predisposing to spasm, should enjoin caution in the use of Emetics. They are apt to excite cramp of the stomach in persons of this habit. The best preventive, is the administration of a large dose of opium, several hours before the emetic; and the union of ipecacuanha and finely powdered valerian root, with the tartarized antimony.

Congestive fevers are frequently much relieved by Emetics; but, if the prostration be very great, they are dangerous remedies, unless internal, and, more particularly, free external stimulation, be employed at the same time.

Emetics are contraindicated by the exhaustion of the vital powers attendant on the latter stages of most diseases, whether acute or chronic; and might sometimes destroy life, by their debilitating operation.

When the feet, or the surface of the body, is cold, and the perspiration suppressed by exposure to a damp atmosphere, Emetics should not be given. Nor should the patient at any time take an emetic in a cold and damp room.

In old age Emetics operate less kindly, than in childhood, and hence they are better adapted to the diseases of early, than latter life.

The best periods of the day for taking an Emetic, are, the morning, before the patient has risen, and the evening, in time for the operation to be over, and some cathartic effect to take place, before going to rest for the night.

Emetics produce drowsiness, and it is a common opinion of the people, that the patient should not be allowed to sleep, till the operation is completed. The only harm that sleeping can do, is to limit or suspend the operation of the medicine, and determine its action on the bowels. A state of wakefulness and an erect position promote the operation of the medicine, and when this is tardy, they should both be enjoined.

Hyper-emesis or excessive vomiting from an emetic, is a true cholera; and may even prove fatal. It is proper, therefore, that those who are in the habit of administeriug this class of medicines, should be familiar with the prophylactic and curative means of meeting such a case. I have elsewhere, (see the number for September, 1827, vol 1, of this Journal,) spoken at large on this subject, but will conclude the present article with a summary of what is requisite in such cases.

Cramp of the stomach, from the action of emetic medicines, presents two stages, which require different methods of treatment. The first is characterized by spasm, the second by inflammation. The former is to be met by pediluvium, and dry epigastric cupping, the application, to the pit of the stomach, of a strong sinapism, a flannel dipped in hot spirit of turpentine, or a towel, wrung out of hot water, rolled into a ball, and enveloped in a dry wrapper. The most efficacious internal remedies, are hot spirit and water, and large doses of laudanum; if the bowels have not been acted on, a purgative injection should be promptly administered; otherwise, an injection of starch with laudanum, will be proper. Should the symptoms continue for several hours, the second or inflammatory stage will be developed, and the treatment should be that for acute gastritis. A perseverance in the stimulating course, would be fatal. The patient should be bled, and cupped or leeched, in the epigastric region;—afterwards, (the symptoms continuing,) blistered. Mild mucilaginous drinks should be given sparingly, and resort at length be had to a pill of calomel and opium, with a repetition of the blood-letting.

As the powers of the system are extremely depressed in this affection, the patient should be kept in a recumbent posture, with his abdominal muscles relaxed, and encouraged to sleep.

*Cincinnati, April* 1, 1832.

